# Pharmacokinetics of Colistin in the Gastrointestinal Tract of Poultry Following Dosing via Drinking Water and Its Bactericidal Impact on Enteric *Escherichia coli*

**DOI:** 10.3389/fvets.2021.698135

**Published:** 2021-06-24

**Authors:** Andrew Mead, Pascal Richez, Stefano Azzariti, Ludovic Pelligand

**Affiliations:** ^1^Comparative Biomedical Sciences, The Royal Veterinary College, London, United Kingdom; ^2^Transpharm, Saint-Genies des Mourgues, France

**Keywords:** polymyxin antibiotics, chicken-broiler, *Eschericha coli*, colistin, pharmacokinetics

## Abstract

Colistin, a last-line antibiotic of major importance in veterinary medicine and of critical importance in human medicine, is authorized to treat gastrointestinal (enteric) infections caused by non-invasive *Escherichia coli* in multiple veterinary species including poultry. Its use in veterinary medicine has been implicated in the widespread prevalence of mobilized colistin resistance. The objectives of this study were to determine the intestinal content reached in broiler chickens during 72-h treatment with colistin, to evaluate the associated impact on intestinal *E. coli* density, and to select less susceptible *E. coli* populations. In this study, 94 broiler chickens were administered a dose of 75,000 IU/kg/day via drinking water. Intestinal samples were collected pre-, during-, and post-dosing. Luminal intestinal content was assessed for colistin content by ultra-high-performance liquid chromatography-tandem mass spectrometry (UHPLC-MS/MS), and *E. coli* were isolated and enumerated on UriSelect agar™. Minimum inhibitory concentration (MIC, for eight isolates per intestine per animal) was determined, and when higher than the epidemiological cutoff (ECOFF 2 mg/l), isolates were screened for mobilized colistin resistance (mcr)-1 to 5. Colistin content increased during treatment to a maximum of 5.09 mg/kg. During this time, the total population of *E. coli* showed an almost 1,000-fold reduction. An apparent increase in the relative abundance of *E. coli* with an MIC ≥ ECOFF, either mcr-negative (6.25–10.94%) or mcr-1-positive (4.16–31.25%) was observed, although this susceptibility shift was not maintained post-treatment. Indeed, following cessation of dosing, colistin was eliminated from the intestine, and content was below the limit of quantification (LOQ, 1.1 mg/kg) within 4 h, and the median MIC of *E. coli* isolates returned below baseline thereafter. Few isolates with a lower susceptibility (mcr-1-positive or negative) were however observed at the end of the study period, indicating maintained sub-populations in the chicken gut. The results of this study show a limited impact on long-term maintenance of less susceptible *E. coli* populations as a direct result of colistin treatment in individual birds.

## Introduction

Colistin, polymyxin E, has been used in veterinary medicine since the early 1950's following its discovery in 1949 from the bacterium *Bacillus polymyxa colistinus* (1, 2). Like other polypeptide antibiotics, colistin is a multi-component compound consisting of over 13 cyclic polypeptides that differ in the length of the fatty acyl segment (3). The two core components are colistin A and B (polymyxin E_1_ and E_2_), which can comprise up to 95% (dependent on source/batch variation) of the overall mass. Due to the inherent variability between colistin batches, dosing needs to be considered in terms of the individual potency (minimum 19,000 IU/mg). To standardize measurement across dosing regimens and studies, the amount of colistin is reported in terms of colistin base, calculated through potency comparison to the international standard set at 30,000 IU/mg. Since its introduction, colistin has been used across all continents in intensive farming practices of multiple species including swine (4), bovine (5), and poultry (6). It has been used not only therapeutically but also as a growth promoter in many countries including, China, India, and Vietnam (7). Many countries have now restricted its use as a feed additive and growth promoter following the discovery of mobilized colistin resistance (*mcr*) genes (8). For example, China banned the use of colistin as a feed additive in 2016 (9). In European Union, indications of use as veterinary drug were restricted in 2015 to therapy or metaphylaxis, all indications for prophylactic use were removed, and indications were restricted to the treatment of enteric infections caused by susceptible non-invasive *Escherichia coli* only (10).

It was thought that resistance to colistin was limited to chromosomal mutations that represented limited vertical transmission within the individual population but posed minimal risk of spread for decades (11). However, the recent discovery of *mcr* elements changed the perspective on colistin resistance as a plasmid-borne transposable element capable of horizontal transfer and rapid spread of colistin resistance. To date, nine major *mcr* variants have been identified in multiple gram-negative species across all continents (12) and represent a global issue.

Colistin use in human medicine, to treat *Enterobacteriaceae* infections, has been limited over the same period due to the inherent nephro- and neurotoxicity of the polymyxins (13). However, an increase in carbapenem-resistant *Enterobacteriaceae* infections has resulted in an increased use of colistin as a last-line antimicrobial to treat these gram-negative infections, and the World Health Organization (WHO) has now listed colistin as an antibiotic of critical importance (14). This has increased scrutiny on the use and dosage regimens in veterinary species and its relationship to the onset and spread of *mcr* (15).

When administered to farmed animals, including poultry, colistin is most often given orally *via* drinking water (16). For the treatment of enteric infection, this is a useful means of administration, as colistin is poorly absorbed from the gastrointestinal tract. Oral treatment results in high concentrations at the site of gastrointestinal infection and minimal risk of antimicrobial residues in the meat associated with a short withdrawal time (17). This form of administration also leads to whole-flock treatment in which the dose is directly related to the amount of water consumed, resulting in varied and potentially suboptimal dosage in a percentage of animals. This study aimed to (i) measure the intestinal content reached during and after a 72-h oral administration *via* drinking water in broiler chickens at the current clinical dose of 75,000 IU/kg (approved in all EU countries for poultry), (ii) to evaluate the bacterial effect on intestinal *E. coli*, and (iii) to track the changes in susceptibility to colistin in commensal and resistant gut *E. coli*.

## Materials and Methods

### Animals and Treatments

This study was approved by the Royal Veterinary College ethics and welfare committee. One hundred Ross 308 broiler chicken (*Gallus gallus domesticus*) were obtained from an approved supplier and housed in the animal welfare barn (AWB) at the Royal Veterinary College. Poultry were group-housed and fed baby chick crumbs (Smallholder Range, Norfolk, UK), a feed free of coccidiostats and designed to feed from hatching to 6–8 weeks, and given free access to water for the duration of the study. At 13 days, six birds were separated and maintained as a control group for the course of the study with the remaining 94 birds constituting the study group. European Pharmacopeia-compliant Meiji Seika Pharma's colistin sulfate (ColiMeiji®, hereafter “colistin”) consisting of a 78.53% (“as is”) mixture of colistin A (polymyxin E1) and colistin B (polymyxin E2), was per certificate of analysis (CoA) supplied by Wyjolab (Chaillac, France). Potency of colistin (as reported by CoA) was 23,558 IU/mg, and dosing was calculated as equivalent colistin base. Colistin was administered *via* drinking water to the study group at a dose equal to 75,000 IU/kg/day over a period of 3 days. The volume of colistin stock solution (2,000,000 IU/ml) diluted per 3-L drinking bell (Poultry Drinker 3L, Farm & Country Supplies, Alton, Hampshire, UK) was based on the average bird weight and water consumption measured on the day prior to dosing. Actual dose received every 24 h was back calculated based on measured water consumption for that period and bird weights. Birds were housed in a 25-m^2^ floor pen with access to 5 × 3-L drinking bells, refreshed every 24 h. Average relative humidity was 44.5% (range: 29–63%) and average temperature 24°C (range: 20–28°C). Birds were provided a 14-h photoperiod aligned with onset of dosing (0 h) at 07:00.

The study group was sampled at predetermined times pre-dose (0 h), during oral dosing (at 12, 36, and 54 h), at cessation of dosing (72 h), and after dosing (73, 74, 76, 80, 84, 96, 120, and 144 h from the onset of dosing). At each time point, eight birds were sacrificed, except at 12, 36, and 54 h where only six birds were sacrificed. After neck dislocation, cloacal swabs were immediately taken, and the whole small intestine was excised after placing three ligatures (one around the duodenum, one on the ileum just proximally to the cecal attachment, and one distally to include the large intestine). All samples were stored at 4°C for no more than 48 h prior to further analysis. All birds in the control group underwent cloacal sampling at matching time points.

### Collection of Luminal and Parietal Intestinal Content

Each intestinal specimen was individually processed with a new set of examination gloves and blades to avoid carry over or contamination. The ceca were separated from the small intestine by cutting off the ileum proximally to the ligature. The luminal intestinal content (LIC) was then evacuated through peristaltic massage of the small intestine in a proximal-to-distal direction. Extracted LIC was mixed to ensure a homogenous sample, and 200 mg (±2 mg) aliquots were separated for further testing. Parietal intestinal content (PIC) was collected following LIC; the intestine was inflated with air, straightened, and longitudinally incised with a scalpel blade no. 11. The opened intestine was flattened, and the blade was used to scrape the parietal lining of the intestine. Luminal cecal content (LCC) was extracted in a similar fashion following incision at one end. Aliquots for colistin quantification were stored at −80°C or at 4°C for bacterial enumeration and isolation.

### Determination of Total Colistin Content in Extracted Intestinal Matrices

Total colistin content was quantified using a novel ultra-high-performance liquid chromatography-tandem mass spectrometry (UHPLC-MS/MS) method. Method validation performed in line with VICH GL49 guidelines indicated limit of quantification (LOQ) of 1.1 mg/kg, accuracy of 13.3% (intra-day)/15% (inter-day), precision of 88.9% (intra-day)/101.3% (inter-day), and both long-term (up to 15 weeks) storage stability and freeze–thaw (three cycles) stability (Mead et al., 2021, personal communication). Briefly, colistin was extracted from 200 mg (±2 mg) of intestinal sample through addition of 1.5 ml extraction solution [methanol/4 M sulfuric acid (1:2; *v*/*v*)]. After adding the internal standard (polymyxin B), the sample was homogenized using a mechanical agitator. The homogenized solution was centrifuged, and supernatant used for solid-phase extraction (SPE) on an Oasis HLB column, which was preconditioned with 5 ml of methanol and rinsed with two washes of 4 ml deionized water. Elution with methanol/formic acid (99.9:0.1; *v*/*v*) followed by evaporation under nitrogen resulted in a dried extract for quantification. The dried extract was reconstituted in water/formic acid (99.9:0.1; *v*/*v*) and used for UHPLC-MS/MS analysis. Separation was performed using UHPLC-MS/MS on an ACQUITY ultra-performance liquid chromatography system with BEH C_18_ separation column (1.7 μm particle size, 2.1 × 50 mm) (Waters, Milford, MA, USA) coupled with a Xevo TQ-S micro Triple Quadrupole Mass Spectrometer (Waters, Milford, MA, USA). Quantification was calculated based on internal standard recovery and matrix-matched external calibration (calibration range: 1.1–28.4 mg/kg; LLOQ: 1.1 mg/kg).

### Enumeration and Isolation of *E. coli*

UriSelect 4™ agar (Bio-Rad, Watford, UK) plates were used for enumeration and isolation of *E. coli* from LIC. Vancomycin (16 mg/ml) was added to inhibit the concomitant growth of *Enterococcus* on this media. An aliquot of 200 mg (±20 mg) of LIC was suspended in 1 ml of phosphate-buffered saline (PBS; Oxoid, Hampshire, UK) and vortexed for 5 min to homogenize. A 10-fold dilution series (from undiluted to 10^−6^) was prepared, and 100 μl from each dilution spread onto agar for overnight incubation at 37°C. Cloacal swabs were moistened with PBS and swabbed directly onto UriSelect 4™ agar for isolation of *E. coli*. Following incubation, *E. coli* were identified as pink colonies according to manufacturer's instruction. Bacterial counts in original LIC were back calculated based on the total number of colonies countable on UriSelect 4™ agar.

For each sample originating from a specific chicken intestine, eight presumptive *E. coli* colonies were selected at random for each LIC sample and sub-cultured to confirm purity. Species identification was later confirmed by PCR, using the protocol described by Le Devendec et al. (18) Isolates were stored in Mueller Hinton Broth (MHB; Oxoid, Hampshire, UK) with 25% glycerol (Fisher Scientific, Loughborough, UK) at −80°C for further analysis.

### Minimum Inhibitory Concentrations

Minimum inhibitory concentration (MIC) was determined for each of eight isolates per bird, with either six or eight birds per time point as previously described. *E. coli* was confirmed in 717 out of 752 LIC samples and in 705 out of 752 cloacal samples. Isolates that could not be confirmed at *E. coli* by PCR were excluded. MIC was determined in confirmed *E. coli* according to the broth microdilution method described in the European Committee for Antimicrobial Testing (EUCAST) guidelines and in accordance with ISO-20776, including two control isolates [mcr-1 negative (NCTC 12241 with expected MIC 0.5 or 1 mg/l) and mcr-1 positive (NCTC 13846 with expected MIC 4 mg/l)] (19). This laboratory work involving handling of the mcr-1-positive control isolate was not carried out at the time of *in vivo* sampling. A twofold dilution series (0.125–64 mg/l) was prepared in cation-adjusted Mueller Hinton Broth (CAMHB; Oxoid, Hampshire, UK) using a semi-automated pipetting system (VIAFLO ASSIST, INTEGRA Biosciences, Thatcham, UK). Bacterial suspensions were prepared from individual colonies suspended in PBS with comparison to 0.5 McFarland standards using DensiCHECK Plus (BioMerieux, Hampshire, UK). Dilution of this suspension was done with CAMHB to achieve a final, in-plate, inoculum of 5 × 10^5^ colony forming units (CFU)/ml. The MIC was recorded following overnight (16–18 h) static incubation at 37°C. Two *E. coli* control isolates were included in each plate. MIC within one dilution of the expected range, to account for possible variation in MIC measurement, confirmed validity of result.

### Resistance Gene PCR Screening

Isolates that are commonly considered as “resistant” from an epidemiological point of view (MIC ≥ 2 mg/l) were screened for the presence of mcr genes (mcr-1, mcr-2, mcr-3, mcr-4, and mcr-5) using a multiplex screening method as described by Rebelo et al. (20). *E. coli-*specific (16S rRNA) primers described by Le Devendec et al. (18) were used as a control in each reaction. Briefly, the reaction parameters were as follows: the reaction mixture consisted of 12.5 μl DreamTaq Green PCR Master Mix (Fisher Scientific, Loughborough, UK), 5.5 μl nuclease-free water (Fisher Scientific, Loughborough, UK), 0.5 μl of each of the 12 primers (10 μM), and 2 μl of DNA template. Thermal lysis of 1 ml of overnight culture at 100°C for 5 min, followed by centrifugation at 13,000 rpm, provided the DNA lysate. The thermal cycler (Techne, Staffordshire, UK) conditions were as follows: 15-min denaturation at 94°C, 25 cycles of 30 s at 94°C, 90 s at 58°C, 60 s at 72°C, and a final elongation at 72°C for 10 min.

PCR amplicons were separated using agarose gel electrophoresis (1.5% agarose; Fisher Scientific, Loughborough, UK). Amplicon sizes were determined against GeneRuler 100 bp DNA Ladder (Fisher Scientific, Loughborough, UK). Controls were included as *E. coli* mcr-1 positive (NCTC #13846), *E. coli* mcr-2 positive (21), *E. coli* mcr-3 positive, *E. coli* mcr-4 positive, and *Salmonella paratyphi* mcr-5 positive (20).

### Statistical Analysis

Viable *E. coli* counts were compared, following log_10_ transformation, using ANOVA followed by *post-hoc* Tukey's multiple-comparison test with GraphPad Prism software (version 8.4.3, GraphPad Software Inc., San Diego, CA, USA). Mean MICs were compared for different time points and sites (intestinal vs. cloacal), following log_2_ transformation, with a repeated measures ANOVA and Fisher's *post-hoc* test in R (version 4.0.3). Values of *p* < 0.05 were taken as statistically significant.

Among *E. coli* strains with MIC ≥ 2 μg/ml, isolates confirmed mcr-1 to mcr-5 were distinguished from the ones with non-identified resistance factors. We evaluated within-chicken correlation with regard to the emergence of isolates with MIC ≥ 2 μg/ml. The association between the presence of mcr-1 within a chicken and the recovery of two or more mcr-1 isolates (vs. a single isolate only) within the same bird's intestinal sample was tested with a chi-square test. Odds ratio for clustering was computed with the Baptista–Pike method.

### Non-compartmental Pharmacokinetic Analysis

Non-compartmental PK analysis (NCA) was carried out with Phoenix WinNonlin 8.2 (Certara, Princeton, NJ, USA). Although birds proportionally drink more during the period of light of the cycle, the dosing was considered as a continuous and monotonous input until the next change of drinking water (7 a.m.). The sparse option of NCA (taking the mean concentration value for each unique time value for LIC data at each nominal time) was used to take into account for the destructive nature of the sampling, in which one bird generated one gut content point. Linear trapezoidal linear interpolation calculation method was used. The mean partial area under the curve (AUC) of digesta were calculated for AUC_0−24*h*_, AUC_0−48*h*_, and AUC_0−72*h*_ as well as the mean colistin intestinal content during these intervals.

## Results

### Back-Calculation of Dose Administered

The average doses actually consumed during each of the 24-h period of administration were back-calculated from the average volume drunk per day (around 40 ml/kg/day) and bird weight (170–200 g) (Table 1). The average doses in water were 66,598; 69,657; and 83,208 IU/kg/day for 0–24, 24–48, and 48–72 h periods, respectively, yielding an average dose of 73,154 IU/kg/day over the duration of the dosing, i.e., very close to the target dose of 75,000 IU/kg/day.

**Table 1:** Colistin sulfate administration and calculated average dose administered^*^.

**Day**	**Water volume drunk per bird over 24 h (mL)**	**Average weight of the sample of chicken (g)**	**Volume of stock solution added to 2 L**	**Actual dose for the day (IU/kg/d)**
Day 1 (0–24 h)	39.69	171.7	0.288 mL	66 598
Day 2 (24–48 h)	40.45	188.3	0.324 mL	69 657
Day 3 (48–72 h)	43.7	196.3	0.374 mL	83 208
Average 0–72 h				73 154

* *The volume of freshly made colistin stock solution (2,000,000 IU/ml) diluted per 2-L drinking bell was based on the average bird weight and water consumption measured on the day prior to dosing. Actual dose received every 24 h was back calculated based on measured water consumption for that period and bird weights*.

### Pharmacokinetics of Colistin

**Figure 1:**
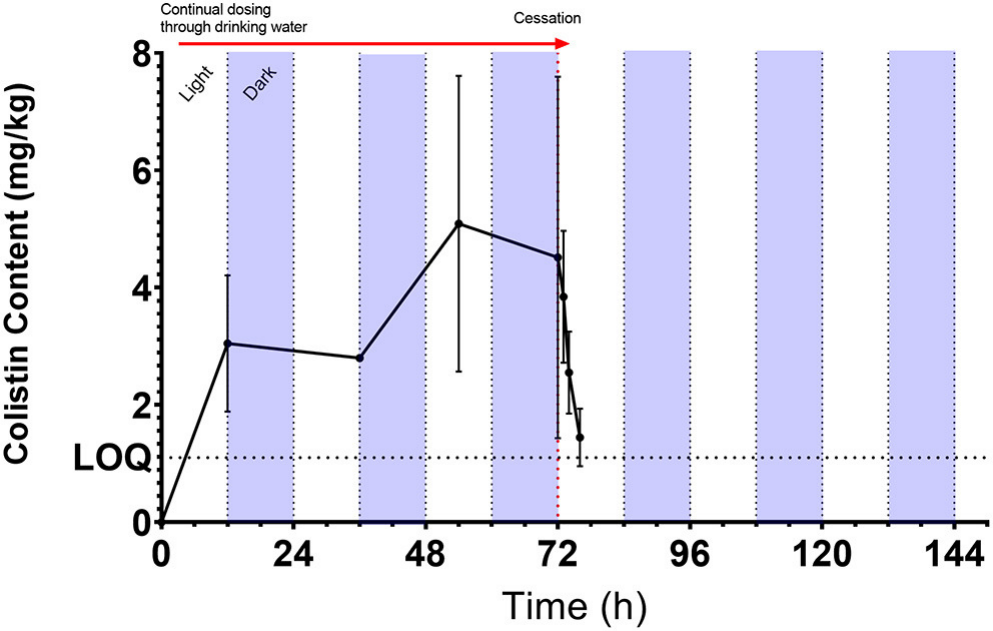
Mean [±SD; *n* = 6 (0, 12, 36, and 54 h) or 8 (72 h onward)] colistin content (mg/kg) as measured by UHPLC-MS/MS in chicken luminal intestinal content during and following administration of 75,000 IU/kg colistin sulfate *via* drinking water. Red arrow denotes period of dosing *via* drinking water from administration of dosing at 7 a.m. Light/dark cycle represents photo- and scotoperiods.

Colistin content in LIC was measurable from the first time point (12 h post-onset of dosing) and was detectable throughout the dosing period, with a consistent increase in mean content up to a peak of 5.09 mg/kg (range: 2.34–9.76 mg/kg) at 54 h. On the morning of the cessation of dosing (72 h), content was 4.51 mg/kg (range: 1.72–10.07 mg/kg) and colistin content declined rapidly, dropping below the LOQ by 76 h (4 h post-withdrawal of colistin administration) (Figure 1). The half-life of disappearance of colistin from the luminal intestinal content was 2.5 h after cessation of dosing. The mean partial AUC of digesta calculated for AUC_0−24*h*_, AUC_0−48*h*_, and AUC_0−72*h*_ periods yielded mean colistin intestinal contents of 2.25, 2.70, and 4.69 mg/kg digesta for these respective intervals (Table 2). Over the course of the 72-h dosing, the average colistin content in LIC was 3.36 mg/kg.

**Table 2:** Partial AUC calculations and average colistin intestinal content (or average colistin content in the digestive tract) after administration of a nominal dose of 75,000 IU/kg/day of colistin sulfate^*^.

**Time period**	**Unit**	Partial AUC (mg/kg^*^h)	**Average colistin luminal intestinal content (mg/kg digesta)**
**AUC**_**0−24h**_ **(first day)**	h^*^mg/kg	**54.1**	**2.25**
AUC_24**–**48h_ (second day)	h^*^mg/kg	75.5	3.15
**AUC**_**0−48h**_ **(first and second day)**	h^*^mg/kg	**129.6**	**2.70**
AUC_48**–**72h_ (third day)	h^*^mg/kg	112.6	4.69
**AUC**_**0−72h**_ **(three days)**	h^*^mg/kg	**242.2**	**3.36**

* *Non-compartmental analysis (sparse option) carried out from six to eight birds destructively sampled per time point. Colistin limit of quantification in LIC by UHPLC-MS/MS was 1.1 mg/kg of digesta*.

*Bold values represent total for 24 h, 48 h, and 72 h respectively. Non-bold are for individual days (E.g. 24–48 h, and 48–72 h). This is clarified in the brackets for each time period*.

Parietal content was not reliably quantifiable at any time points, with the exception of one bird at each of the 12- and 54-h (during dosing) and 72-, 73-, and 74-h time points (0, 1, and 2 h post-dosing). These five quantifiable samples ranged from 1.04 to 2.55 mg/kg, and their content was, on average, 3.51-fold lower than the corresponding luminal intestinal content. Colistin was only measurable in six cecal content samples and detectable (<1.1 mg/kg LOQ) in seven further samples. The cecal content of the six quantifiable samples was, on average, 3.01-fold lower relative to the corresponding luminal intestinal content.

### Effect of Colistin on Enteric *E. coli* Enumeration

**Figure 2:**
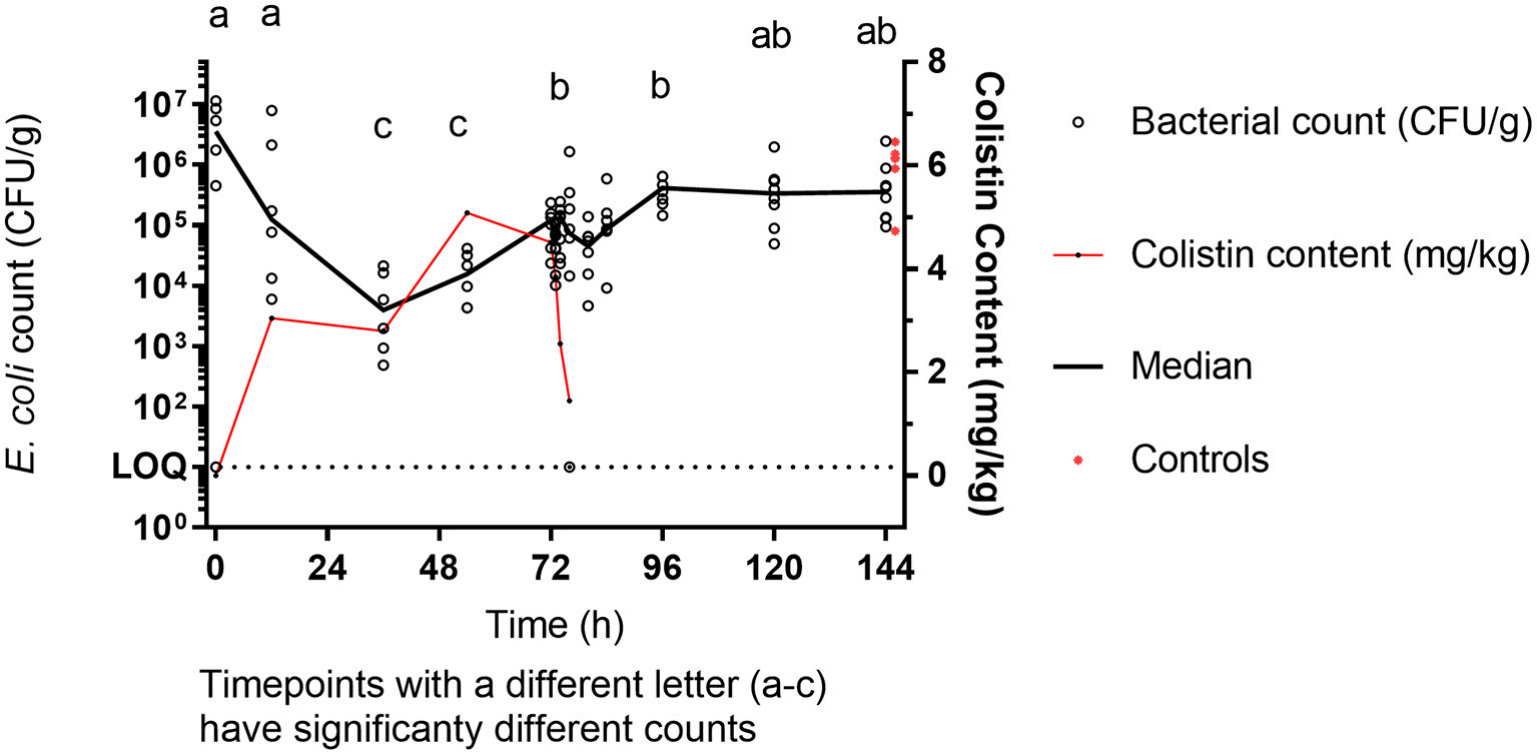
*E. coli* enumeration (CFU/g) vs. mean [±SD; *n* = 6 (0, 12, 36, and 54 h or 8 (72 h onward)] colistin content (mg/kg) in luminal intestinal content during colistin sulfate administration at clinical dose. Letters **(a–c)** represent significantly different counts.

The median baseline *E. coli* count was 3.6 × 10^6^ CFU/g. During colistin sulfate administration, a significant reduction in *E. coli* count by a factor of 1,000 to 100 was obtained at 36 and 54 h (median count 3.9 × 10^3^ CFU/g and 1.56 × 10^4^ CFU/g, respectively, *p* < 0.05) compared to baseline. By 72 h, *E. coli* count was 1.46 × 10^5^ CFU/g, still lower than baseline counts but significantly higher (*p* < 0.05) than the 36-h trough (Figure 2). *E. coli* counts were similar at 96, 120, and 144 h, in the region of median 5 × 10^5^ (end of the experiment) and like those from the untreated birds terminally taken at 144 h (median 1.29 × 10^6^ CFU/g). This final count was significantly different from the trough numbers of *E. coli* counted during treatment (36 h, *p* < 0.05), but not significantly different from the original baseline.

### Effect of Colistin on Enteric *E. coli* Susceptibility

Enteric *E. coli* isolates ranged from 0.125 to 16 mg/l with a MIC_90_ of 2 mg/l. Due to the range of the assay, the MIC distribution was left censored at 0.125 mg/l with this dilution accounting for 487/717 of the MICs. Overall, 97 out of the 1,469 isolates collected showed phenotypic colistin epidemiological resistance (MIC ≥ 2 mg/l). Of these, 48 were shown to harbor mcr-1. All isolates were negative for mcr-2, mcr-3, mcr-4, and mcr-5, indicating an undefined mechanism of decreased susceptibility in the remaining isolates (Figure 4).

**Figure 3:**
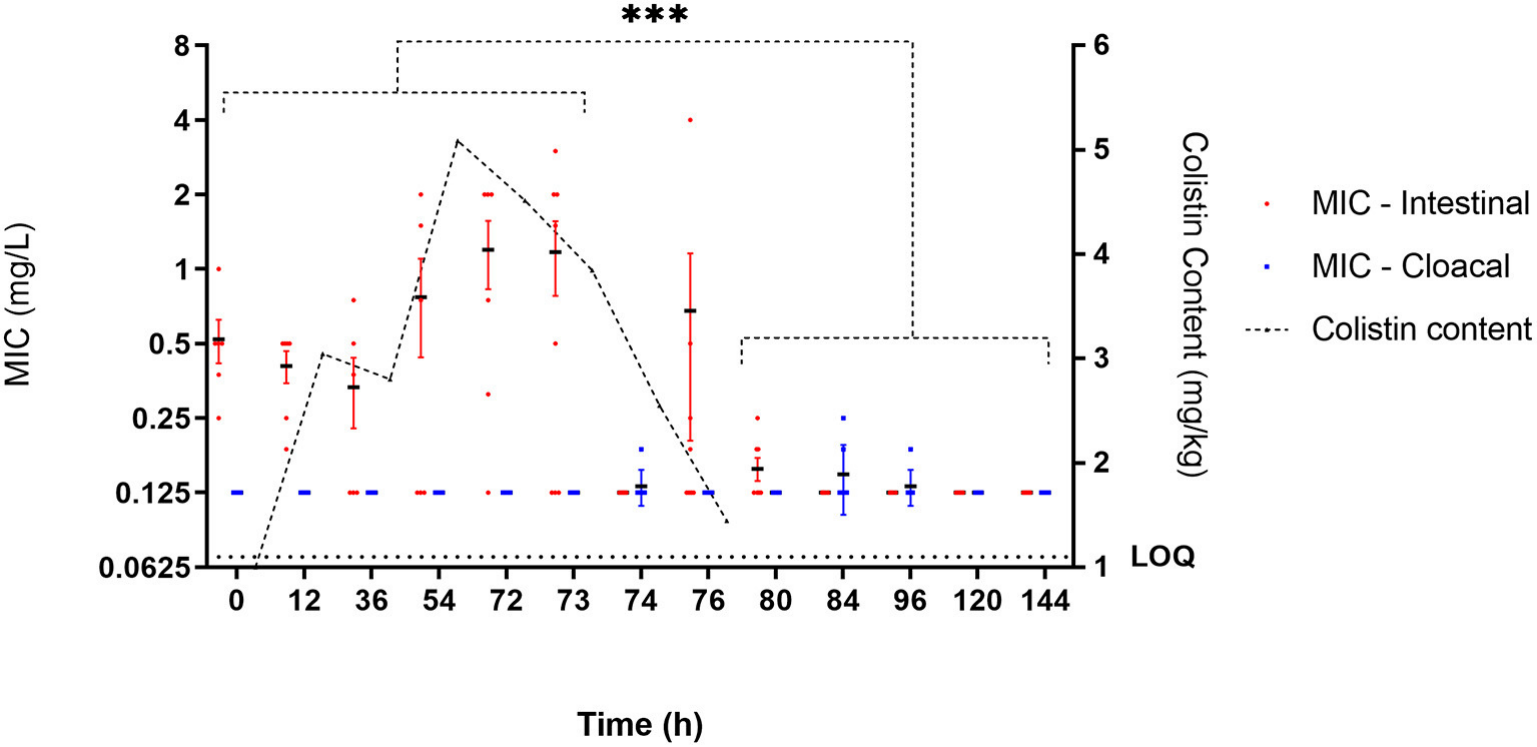
*E. coli* susceptibility as measured by MIC (mg/l) during administration of colistin sulfate at clinical dose (75,000 IU/kg). Each individual point represents the mean MIC of isolates (*n* = 8) for an individual bird. Black horizontal bar represents mean of all birds at that time point (*n* = 6 or 8). Red and blue error bars represent SEM for intestinal and cloacal samples, respectively. Significance represents intestinal isolates only, and cloacal isolates are not considered representative of digestive bacterial population. ***Significant at *p* < 0.001.

**Figure 4:**
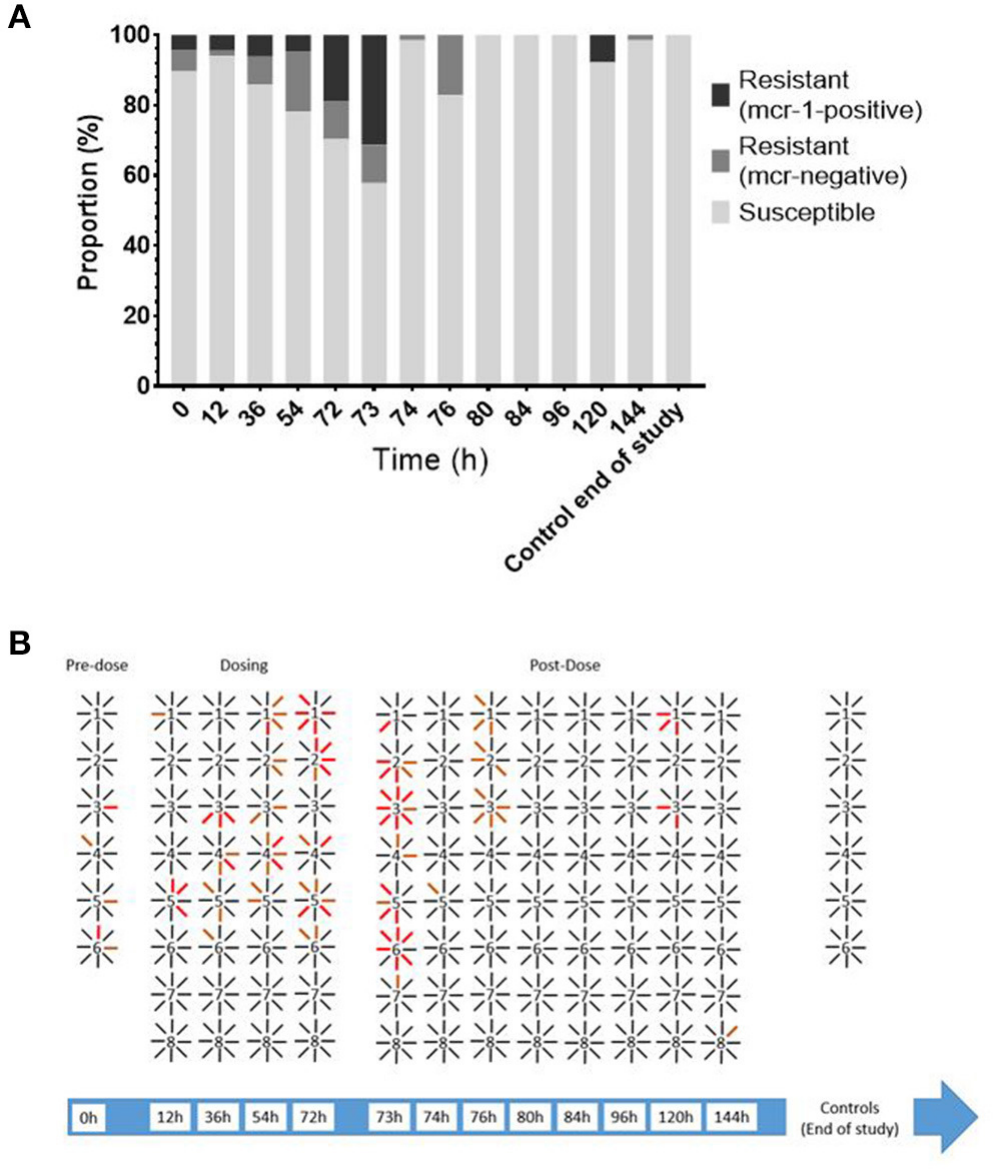
**(A)** Proportion of *E. coli* isolates from LIC [*n* = 48 (0, 12, 36, and 54 h) or 64 (72 h onward)] susceptible, resistant (MIC ≥ 2 mg/l), and mcr-1-positive isolates over time. **(B)** Filiation between bird (number) and individual isolates recovered (lines). Black line = susceptible; orange line = resistant mcr-negative; red line = resistant mcr-1-positive.

Prior to colistin exposure (baseline 0 h), the average MIC (arithmetic mean of eight isolates) for the six chickens ranged from 0.25 to 1 mg/l (Figure 3). Of these 48 baseline isolates, there were five isolates with MICs equal to or higher than the epidemiological cutoff value (ECOFF) of 2 μg/ml (10.42%). Two of them were mcr-1 positive (4.17% of all isolates) (Figure 4).

During exposure to colistin (from the 12 to 72-h time points), the average MIC within chicken increased, with specific isolates reaching MICs of up to 4 mg/l, though this was not significant when compared to the baseline level. At time point 73 h, the maximum number of isolates showing phenotypic epidemiological resistance to colistin was 21 of 41 isolates (51.22%), of which 16 where mcr-1 positive (39% of all isolates).

After the cessation of treatment (post 72 h), the average MICs within chicken were significantly (*p* < 0.01) below baseline (most of them were 0.125 mg/l) with only five isolates with MIC ≥ 2 μg/ml observed at 120 h and only a single isolate with lower susceptibility at end of the study (Figure 3). The geometric mean MIC within chicken from the control group was 0.14 μg/ml, and 100% of the 48 isolates recovered from this untreated group had an MIC below 2 μg/ml.

There was a statistically significant difference within chicken correlation or clustering, with regard to the distribution of epidemiologically resistant strains (*p* < 0.046, chi-square). When at least one mcr-1-bearing isolate was identified within a chicken (out of eight isolates), it was 2.8 times more likely (95% CI 1.12–6.44) to have originated from a sample with several mcr-1 isolates rather than from a sample with single mcr-1 isolate.

### Effect of Colistin on Cloacal *E. coli* Susceptibility

MICs of cloacal *E. coli* isolates ranged from 0.125 to 2 mg/l with a MIC_90_ of 2 mg/l, although were left censored at 0.125 mg/l (644/707 cloacal isolates). Cloacal MICs were 0.125 mg/l with average MIC increasing modestly at discrete late time points (74, 84, and 96 h). Throughout the time course, no colistin isolates with MIC > 2 mg/l were identified.

## Discussion

In this study, the PK profile of colistin was determined in the small intestine of chickens during a 3-day period of colistin sulfate administration at 75,000 IU/kg and 3 days post-dosing. The impact of this dosing regimen on enteric *E. coli*, a key source of pathogen of colibacillosis and production loss in poultry, is demonstrated and the selection of resistance patterns explored. To date, there has been a single study describing the pharmacokinetics of colistin in the poultry gut digesta (22). However, this was after oral gavage of 25 and 50 mg/kg doses, at least 10-fold the current clinical dose of 75,000 IU/kg/day (2.5 mg colistin base equivalent/kg/d) in drinking water.

Sato et al. (22) showed that colistin is detectable in the small intestine within 2 h of oral gavage. In our study, following administration *via* drinking water, a rapid increase in colistin concentration within the luminal intestinal content was observed by 12 h, but we did not have an earlier sampling point to demonstrate that colistin reached the small intestine earlier. Using water administration, the consumption of water is assumed to be comparable between chickens during a given time period. We only measured group 24-h water consumption and did not video-track individual chicken, but there was evidence of variability in intestinal content between birds (Figure 1). This could reflect inter-animal variability in the amount or timing of water uptake during the day/penumbra/night continuum.

At cessation of dosing, 72 h from start of administration, colistin was rapidly cleared from the small intestine, dropping below the LOQ between 4 and 6 h. Svihus et al. (23) monitored the transit time in broiler chickens using the passage of titanium dioxide and identified that a significant amount had passed through the small intestine within 2.5 h. Differences in feed type, bird size, and age may have contributed to the difference between their observations and the elimination of colistin in this study. The time for colistin to drop below the limit of quantification in this study more closely resembles that observed by Rougière and Carré (24) and Ravindran (25) who reported a transit time of 3.82–5.65 h and 4.6 h, respectively. The key factor affecting colistin elimination through the small intestine is the peristaltic flow through the broiler gut; no residual colistin was maintained within the cecum, despite the reported longer transit time in this organ (26) and the existence of retro-peristaltic flow (27) within the intestinal wall.

The increase in intestinal colistin content correlates with a decrease in the number of viable *E. coli*, within the luminal intestinal matrix, up to 36 h post-onset of colistin administration. During this period, a >3-log reduction (99.9%) in the number of *E. coli* was observed, indicating that colistin has an initial bactericidal effect with mean colistin content reached within the first 24 h was around 2.8 mg/kg. The unit conversion between intestinal content (mg/kg) and antimicrobial potency (mg/l) is yet to be confirmed, as it may depend on the sorption of colistin to foodstuff, the density of intestinal content, the local pH, and the capacity of bacteria to replicate in gut (which could be substantially lower than in standardized culture broth). Intestinal microdialysis experiment could refine this estimate, as performed by Foster et al. (28) in calves, but was not feasible in such small birds. However, assuming a 1 mg/kg content:0.8 mg/l ratio conversion (29), the antimicrobial potency of luminal colistin exceeded the baseline MICs (0 h) and the ECOFF (2 mg/l) reported by EUCAST, thus explaining the bactericidal effect. Viel et al. (30) reports fecal concentrations of 93 μg/kg having bactericidal effect of *E. coli* after 1 day of oral administration in pigs at the dose of 100,000 IU/kg/day, but the product was given by gavage, not through continuous access to medicated drinking water.

The intestinal content over the entire treatment period was maintained around 2.69 mg/kg in our birds treated with 75,000 IU/kg/day; however, the remaining *E. coli* population was able to recover after 36 h. The regrowth event, associated with an apparent increase in MIC, may be attributed to a selective pressure, asserted by the presence of colistin, on less susceptible isolates present within the intestinal tract. In any bacterial population comprising predominant population susceptible to a given antibiotic, there will be sub-dominant populations of genetically different and potentially more resistant bacteria (29). This observation confirms that *E. coli* strains susceptible to colistin were inhibited or killed during treatment due to the high concentrations (exceeding usual MIC levels) reached in the gastrointestinal tract, leaving space for strains that were less susceptible (e.g., with MIC of 4–8 μg/ml). It should be noted however that a reduction in susceptible *E. coli* populations inherently skews the selection of less susceptible isolates during sample analysis.

This indicates that the current clinical dose is able to exert a bacteriostatic/bactericidal effect on wild-type isolates with low MICs but is unlikely to have an effect on sub-populations with higher MICs. It should also be considered that absorption of colistin from the gastrointestinal tract is extremely low. Sato et al. (22), providing a dose of 750,000 IU/kg as an oral gavage, a dose 10-fold in excess of the normal clinical dose (75,000 IU/kg), were only able to transiently measure colistin in serum for 2 h, with a peak of 1.5 μg/ml, and could not quantify it in edible tissues. A limitation of our study was not being able to measure serum concentrations, but it is considered that the clinical dose *via* drinking water would have no attained blood and tissue levels adequate to treat common extra-digestive signs of colibacillosis in poultry, i.e., localized (e.g., omphalitis) or systemic infection (i.e., colisepticemia) (31). The virtually nil absorption and tissue distribution of colistin after oral administration has largely been demonstrated by CVMP in its assessment of residues in food animals (EMEA/MRL/812/02-FINAL).

Regrowth has been previously described in relation to antibiotic treatment, including for polymyxins (32). Studies exploring the transitory adaptive phenotypic resistance to polymyxins indicate that short-time, and culturally unstable, heterogenicity of phospholipids results in susceptibility shifts that promote regrowth even in the continued presence of polymyxin (33). Although the initial bacterial counts did not recover 3 days after treatment withdrawal to baseline level, the wild-type and susceptible population had almost re-colonized the gastrointestinal tract at that time. Any indications of this transitory adaptive resistance had likewise been lost post-treatment, and the remaining population seemed to have a somewhat increased susceptibility to colistin. These results, together with the apparent decrease in total bacterial counts during the treatment period and lack of resistant isolates from cloacal swabs, fail to show any notable increase in the number of resistant strains eliminated in feces. Therefore, it cannot be concluded that the environment was exposed to a notably higher number (absolute value) of *E. coli* strains resistant to colistin; it is also considered that cloacal samples are not a suitable surrogate for intestinal samples.

To further explore the extent of colistin impact on enteric *E. coli* populations, these experiments should be reproduced with different infra- and supra-clinical doses, alternative modes of administration including oral gavage and pulse dosing, and comprehensive monitoring of *E. coli* populations *via* enumeration and susceptibility testing.

MCR has become a widespread global issue and has often been reported in relation to the use of colistin in intensive farming practices (34–36). Recent observations have also shown a marked reduction in mcr-1 carriage following a ban on colistin use as growth promoter in China (37). We screened for mcr within this study to explore the disconnect between gut content and *E. coli* recovery and, to our surprise, did show the presence of mcr-1 carriage in this poultry population. This was identified as a relatively low percentage of isolates and is expected to make up a sub-population within a small number of birds. This differs from the experimental challenge model consisting of three inoculations of 10^7^ CFU/ml of mcr-1 *E. coli* (30) and was not resultant from contamination by lab-based strains due to control measures implemented in the experimental design. A mcr-1 population was present in the birds at the onset of the study, either *in ovo*, on hatch, or from transport. This may indicate the carriage of mcr-1 in UK farms and hatcheries. A study by Viel et al. (30) in pigs also explored the relationship between colistin treatment and mcr-1 selection at clinical and supra-clinical doses and like this study indicated no long-term selection of mcr-1 positive *E. coli*. Many antibiotic resistance mechanisms impose a fitness cost on the bacteria, and in the case of mcr-1, this has been shown to include a reduction in fitness and growth capacity and a loss of virulence (38). It is proposed that in the absence of selective pressure, these mcr-1-positive bacteria are readily out-competed by the otherwise dominant wild-type populations and that proper flock management may provide a means to prevent the spread of mcr-1 in instances where colistin treatment has been used.

This study has limitations as we did not sample the environment for the persistence of mcr-1 (but the birds did not seem to re-contaminate themselves from it). We did investigate whether the growth of the mcr-1 proportion was through clonal expansion or plasmid transfer. Finally, the conversion of colistin intestinal content (mg/kg) into a concentration of active colistin (mg/l) in LIC remains to be determined.

## Conclusion

In summary, the administration of 75,000 IU/kg/day to broiler in drinking water resulted in an initial decrease in the *E. coli* count within the intestinal lumen associated with average colistin content of 3 mg/kg. Treatment reduced the highly susceptible *E. coli* population by a factor of 100 to 1,000, providing an opportunity for the less susceptible sub-population to expand during continuous exposure, until after few hours post-withdrawal when wild-type flora has re-colonized the gut content. Rapid loss of adaptive and mcr-1-positive isolates post-treatment poses a limited risk, provided appropriate animal management, on increasing local environmental resistance burden to colistin.

## Data Availability Statement

The raw data supporting the conclusions of this article will be made available by the authors, without undue reservation.

## Ethics Statement

The animal study was reviewed and approved by Royal Veterinary College ethics and welfare committee.

## Author Contributions

AM, PR, and LP contributed to the conception and design of the study. AM organized, performed all aspects of the study, statistical analysis, and wrote the first draft of the manuscript. SA assisted in performing laboratory analysis. LP wrote sections of the manuscript. All authors contributed to the manuscript revision, read, and approved the submitted version.

## Conflict of Interest

The funding for this study was provided through TransPharm on behalf of Dopharma, V.M.D. Liverstock pharma, and Virbac. PR (TransPharm) was involved in the study design and revision of the submitted article. The funders were not involved in the collection, analysis, and interpretation of data; the writing of this article; or the decision to submit it for publication. AM PhD stipend was covered by the Bloomsbury studentship (see Funding). The remaining authors declare that the research was conducted in the absence of any commercial or financial relationships that could be construed as a potential conflict of interest.
